# Perioperative Non-Invasive Indocyanine Green-Clearance Testing to Predict Postoperative Outcome after Liver Resection

**DOI:** 10.1371/journal.pone.0165481

**Published:** 2016-11-03

**Authors:** Stefanie Haegele, Silvia Reiter, David Wanek, Florian Offensperger, David Pereyra, Stefan Stremitzer, Edith Fleischmann, Christine Brostjan, Thomas Gruenberger, Patrick Starlinger

**Affiliations:** 1 Department of Surgery, Medical University of Vienna, General Hospital, Vienna, Austria; 2 Department of Anesthesiology, Medical University of Vienna, General Hospital, Vienna, Austria; 3 Department of Surgery I, Rudolfstiftung Hospital, Vienna, Austria; Istituto Mediterraneo per i Trapianti e Terapie ad Alta Specializzazione, ITALY

## Abstract

**Background:**

Postoperative liver dysfunction may lead to morbidity and mortality after liver resection. Preoperative liver function assessment is critical to identify preexisting liver dysfunction in patients prior to resection. The aim of this study was to evaluate the predictive potential of perioperative indocyanine green (ICG)-clearance testing to prevent postoperative liver dysfunction and morbidity using standardized outcome parameters in a routine Western-clinical-setting.

**Study Design:**

137 patients undergoing partial hepatectomy between 2011 and 2013, at the general hospital of Vienna, were included. ICG-clearance was recorded one day prior to surgery as well as on the first and fifth postoperative day. Postoperative liver dysfunction was defined according to the International Study Group of Liver Surgery and evaluation of morbidity was based on the Dindo-Clavien classification. Statistical analyses were based on non-parametric tests.

**Results:**

Preoperative reduced ICG—plasma disappearance rate (PDR) as well as increased ICG—retention rate at 15 min (R15) were able to significantly predict postoperative liver dysfunction (Area under the curve = PDR: 0.716, P = 0.018; R15: 0.719, P = 0.016). Furthermore, PDR <17%/min. or R15 >8%, were able to accurately predict postoperative complications prior to surgery. In addition to this, ICG-clearance on postoperative day 1 comparably predicted postoperative liver dysfunction (Area under the curve = PDR: 0.895; R15: 0.893; both P <0.001), specifically, PDR <10%/min or R15 >20% on postoperative day 1 predicted poor postoperative outcome.

**Conclusion:**

PDR and R15 may represent useful parameters to distinguish preoperative high and low risk patients in a Western collective as well as on postoperative day 1, to identify patients who require closer monitoring for potential complications.

## Introduction

Substantial technical and anesthesiological advances in the field of liver surgery now allow performing extended resections also in borderline resectable patients [1]. The most important factor determining postoperative outcome after liver resection represents the ability of the remnant liver to recover [1, 2]. If hepatic regeneration is impaired, postoperative liver dysfunction (LD) occurs, which is associated with a markedly increased risk of postoperative morbidity and mortality. Therefore, a balance between the residual and the resected liver volume is needed to minimize the risk of postoperative LD [2–4].

Therefore numerous methods have been developed to accurately predict poor postoperative hepatic recovery and to determine the maximum amount of liver tissue that can be resected during liver surgery. While volumetry is frequently used to determine future liver remnant, functional assessment of the liver parenchyma is of crucial importance to identify patients that will need an extended amount of remnant liver tissue to sustain postoperative liver function. Accordingly, different quantitative and qualitative tests to assess liver function have been developed [5–9]. Indocyanine green (ICG)–clearance test has been shown to correlate with liver function and has recently been found to predict portal hypertension [10–12].

ICG—clearance testing is a simple, reproducible and non-invasive test. It is routinely performed at the patient's bedside using pulse spectrophotometry. Moreover, ICG is a water-soluble dye that, once injected, selectively binds to plasma proteins. It is further taken up by hepatocytes immediately after binding and is excreted into the bile. It is neither metabolized nor undergoes enterohepatic recirculation. ICG plasma disappearance rate (PDR) as well as the retention rate at 15min. (R15) are evaluated by this method to quantify liver function [13].

While several studies have addressed the predictive value of preoperative ICG-clearance to predict postoperative LD, most of these studies were not based on a standardized definition of LD or morbidity [13–15]. In particular, Cariono et al. and Akita et al. found that ICG—clearance testing significantly correlated with postoperative LD [13–14]. However, both of these studies did not use a standardized classification to define postoperative LD.

Furthermore, little is known about the relevance of ICG-clearance testing in a Western setting, known to differ in terms of underlying disease as compared to an Eastern population [8, 13].

In this study, we aimed to determine the potential of preoperative ICG-clearance testing to predict postoperative LD according to the standardized criteria of the International Study Group of Liver Surgery (ISGLS), in a Western setting. Furthermore, we aimed to evaluate if ICG-clearance was also vital to predict postoperative morbidity according to the Dindo-Clavien classification. Ultimately, the relevance of postoperative ICG-clearance testing to identify patients that will suffer from postoperative LD or morbidity, early after partial hepatectomy, was evaluated. PDR and R15 were compared in terms of their predictive value for postoperative clinical outcome.

## Patients and Methods

### Study Cohort

A total of 137 consecutive patients undergoing liver resection between February 2011 and July 2013, at the general hospital of Vienna, were included in this study. Patients with different neoplastic entities were evaluated, specifically metastatic colorectal cancer (N = 59), hepatocellular carcinoma (N = 30), cholangiocellular carcinoma (N = 23), non-neoplastic diseases (N = 13) and other tumor entities (N = 12). ICG-clearance testing was performed 1 day prior to surgery (Pre OP) and one day (POD 1) as well as five days (POD 5) after liver resection. 137 patients were included in the perioperative- and 126 patients (11 were not available for the postoperative ICG-clearance evaluation) in the postoperative ICG-value measurement. Furthermore, specific characteristics of all patients were prospectively recorded and are compared according to the subsequently defined ICG-clearance cut-off values (Tables 1 and 2). The extent of resection was classified according to the IHPBA Brisbane 2000 nomenclature [16] (<3 segments = minor, ≥3 segments = major). Prothrombin time (PT) is expressed in relation to the coagulation time of a healthy person (i.e. Quick). Accordingly, it is illustrated as the percentage of the normal value of the healthy population.

**Table 1:** Pre-OP Cut-Off-Levels (PDR & R15), Patient demographics, outcome- and laboratory parameters.

Parameter	Collective (N = 137)						
	Pre OP	PDR ≥ 17%/min.	PDR < 17%/min.	P—value	R15 ≤ 8%	R15 > 8%	P—value
	N = / Median (Range/ %)	N = / Median (Range/ %)			N = / Median (Range/ %)		
**Age**	63 (22–89)	62 (22–84)	69 (40–89)	**0.005**	62 (22–89)	67 (40–86)	0.12
**Sex**							
Male	88 (64.2%)	67 (63.8%)	21 (65.6%)	0.85	69 (63.3%)	19 (67.9%)	0.65
Female	49 (35.8%)	38 (36.2%)	11 (34.4%)		40 (36.7%)	9 (32.1%)	
**Neoplastic entity**							
mCRC	59 (43.1%)	43 (41.0%)	16 (50%)	0.05	42 (38.5%)	17 (60.7%)	0.10
HCC	30 (21.9%)	19 (18.1%)	11 (34.4%)		23 (21.1%)	7 (25.0%)	
CCC	23 (16.8%)	19 (18.1%)	4 (12.5%)		20 (18.3%)	3 (10.7%)	
Non neoplastic Adenomas or Haemangiomas	13 (9.5%)	13 (12.4%)	0 (0,0%)		13 (11.9%)	0 (0.0%)	
Others	12 (8.8%)	11 (10.5%)	1 (3.1%)		11 (10.1%)	1 (3.6%)	
**Postoperative CTx**							
yes	51 (37.2%)	40 (38.1%)	11 (34.4%)	0.792	40 (36.7%)	11 (39.3%)	0.70
**Hepatic resection**							
Major	60 (43.8%)	46 (76.7%)	14 (43.8%)	0.10	50 (45.9%)	10 (35.7%)	0.33
Minor	77 (56.2%)	59 (56.2%)	18 (56.3%)		59 (54.1%)	18 (64.3%)	
**LD**							
yes	11 (8%)	5 (4.8%)	6 (18.8%)	**0.011**	6 (5.5%)	5 (17.9%)	**0.032**
**Child Pugh Score/ Missing values (N = 19)**							
A	109 (92.4%)	83 (93.3%)	26 (89.7%)	0.53	86 (93.5%)	23 (88.5%)	0.40
B	9 (7.6%)	6 (6.7%)	3 (10.3%)		6 (6.5%)	3 (11.5%)	
MELD Score	6 (6–16)	6 (6–16)	7 (6–15)	**0.014**	6 (6–16)	7 (6–15)	**0.047**
**Preoperative parameters**							
PDR%/min.	22.1 (7.6–42.8)	24.0 (17.3–42.8)	15.9 (7.6–17.0)		24.0 (16.0–42.8)	15.30 (7.6–29.5)	
R15%	3.6 (0.0–32.0)	2.5 (0.0–9.1)	9.2 (1.2–32.0)		2.6 (0.0–8.0)	9.80 (8.2–32.0)	
SB mg/dl	0.6 (0.2–6.6)	0.6 (0.3–2.8)	0.7 (0.2–6.6)	0.41	0.6 (0.3–3.2)	0.6 (0.2–6.6)	0.85
PT% ^a^	102 (0–150)	103 (0–147)	89 (40–150)	0.65	103 (0–147)	101 (40–150)	0.79
ALP U/l	86.0(14.0–946.0)	84.5 (14.0–946.0)	92.5 (43.0–396.0)	0.47	85.0 (14.0–946.0)	91.0 (43.0–418.0)	0.51
GGT U/l	58.0 (11.0–699.0)	58.0 (11.0–670.0)	59.5 (18.0–699.0)	0.21	54.0 (11.0–670.0)	73.0 (19.0–699.0)	0.10
AST U/l	29 (14–208)	28 (14–113)	33 (18–208)	0.08	28 (14–946)	32 (18–208)	0.12
ALT U/l	26.0 (6.0–196.0)	27.0 (6.0–167.0)	25.5 (13.0–196.0)	0.34	26.0 (6.0–196.0)	27.5 (17.0–120.0)	0.15
Albumin g/l	42.2 (32.5–52.4)	42.5 (33.1–52.4)	41.0 (32.5–46.9)	**0.037**	42.4 (33.1–52.4)	41.0 (32.5–46.9)	**0.038**
Platelets (x10^3/μl)	226.5 (92.0–492.0)	236.0 (92.0–492.0)	213.0 (103.0–378.0)	**0.033**	236.0 (92.0–492.0)	207.5 (132.0–431.0)	**0.036**
Creatinine mg/dl	0.9 (0.5–1.9)	0.8 (0.5–1.9)	0.9 (0.6–1.8)	0.49	0.9 (0.5–1.9)	0.8 (0.6–1.8)	0.13
INR	0.9 (0.7–1.8)	0.9 (0.7–1.6)	1.0 (0.7–1.8)	0.39	0.9 (0.7–1.6)	1.0 (0.7–1.8)	0.89

ALP, alkaline phosphatase; ALT, alanine amino transferase; AST, aspartate amino transferase; CCC, cholangiocellular carcinoma; CTx, chemo therapy; GGT, gamma glutamyl transferase; HCC, hepatocellular carcinoma; INR, international normalized ratio; LD, liver dysfunction; mCRC, metastatic colorectal cancer; MELD, model of end-stage liver diseases, PDR, plasma diffusion rate; pre OP, preoperative; PT, prothrombin time; R15, retention rate at 15 minutes; SB, serum bilirubin

^a^ PT is expressed in relation to the coagulation time of a healthy person (i.e. Quick). Accordingly, it is illustrated in percentage.

**Table 2:** POD 1 Cut-Off-Levels (PDR & R15), Patient demographics, outcome- and laboratory parameters.

Parameter	Collective (N = 137)/ Missing values (N = 11)						
	POD 1	PDR ≥ 10%/min.	PDR < 10%/min.	P—value	R15 ≤ 20%	R15 >20%	P—value
	N = / Median (Range/ %)	N = / Median (Range/ %)			N = / Median (Range/ %)		
**Age (years)**	62 (22–89)	62 (22–89)	62 (39–81)	0.64	62 (22–89)	63 (39–81)	0.38
**Sex**							
Male	80 (63.5%)	74 (64.3%)	6 (54.5%)	0.52	72 (64.3%)	8 (57.1%)	0.60
Female	46 (36.5)	41 (35.7%)	5 (45.5%)		40 (35.7%)	6 (42.9%)	
**Neoplastic entity**							
mCRC	54 (42.9%)	52 (45.2%)	2 (18.2%)	0.25	51 (45.5%)	3 (21.4%)	0.21
HCC	26 (20.6%)	21 (18.3%)	5 (45.5%)		20 (17.9%)	6 (42.9%)	
CCC	21 (16.7%)	19 (16.5%)	2 (18.2%)		18 (16.1%)	3 (21.4%)	
Non neoplastic Adenomas or Haemangiomas	13 (10.3%)	12 (10.4%)	1 (9.1%)		12 (10.7)	1 (7.1%)	
Others	12 (9.5%)	11 (9.6%)	1 (9.1%)		11 (9.8%)	1 (7.1%)	
**Preoperative CTx**							
Yes	48 (38.1%)	46 (40.0%)	2 (18.2%)	0.15	45 (40.2%)	3 (21.4%)	0.17
**Hepatic resection**							
Major	55 (43.7%)	45 (39.1%)	10 (90.9%)	**0.001**	42 (37.5%)	13 (92.9%)	**0.001**
Minor	71 (56.3%)	70 (60.9%)	1 (9.1%)		70 (62.5%)	1 (7.1%)	
**LD**							
Yes	10 (7.9%)	5 (4.3%)	5 (45.5%)	**0.001**	4 (3.6%)	6 (42.9%)	**0.001**
**Child Pugh Score/ Missing values (N = 29)**							
A	99 (91.7%)	91 (93.8%)	8 (72.2%)	**0.016**	89 (93.7%)	10 (76.9%)	**0.040**
B	9 (8.3%)	6 (6.2%)	3 (27.3%)		6 (6.3%)	3 (23.1%)	
MELD Score	6 (6–16)	6 (6–16)	6 (6–15)	0.77	6 (6–16)	6 (6–15)	0.91
**Postoperative Parameters**							
PDR%/min.	20.1 (6.8–72.3)	21.30 (10.1–72.3)	8.9 (6.8–9.8)		21.7 (11.0–72.3)	9.5 (6.8–14.3)	
R15%	5.0 (0.0–36.1)	4.20 (0.0 22.7)	26.30 (23.0–36.1)		4.1 (0.0–19.2)	24.0 (20.7–36.1)	
SB mg/dl	1.2 (0.4–8.2)	1.13 (0.4–6.9)	2.9 (1.0–8.2)	**0.001**	1.13 (0.4–6.9)	2.7 (1.0–8.2)	**0.001**
PT% ^a^	56.0 (29.0–91.0)	57.0 (30.0–90.0)	44.0 (29.0–91.0)	**0.003**	57.0 (30.0–90.0)	45.5 (29.0–91.0)	**0.006**
ALP U/l	60.0 (31.0–335.0)	60.0 (31.0–286.0)	56.0 (40.0–335.0)	0.94	60.0 (31.0–286.0)	63.5 (40.0–335.0)	0.77
GGT U/l	59.0 (6.0–431.0)	58.0 (6.0–335.0)	62.0 (22.0–431.0)	0.47	58.0 (6.0–335.0)	67.5 (22.0–431.0)	0.31
AST U/l	342.5 (56.0–4298.0)	341.0 (56.0–2093.0)	419.0 (269.0–4298.0)	0.16	339.5 (56.0–2093.0)	415.0 (159.0–4298.0)	0.18
ALT U/l	341.0 (70.0–3409.0)	339.0 (70.0–1769.0)	363.0 (164.0–3409.0)	0.56	340.0 (70.0–1769.0)	360.5 (158.0–3409.0)	0.70
Albumin g/l	29.8 (19.6–40.3)	29.9 (19.6–40.3)	28.9 (20.0–34.2)	0.15	30.0 (19.6–40.3)	27.8 (20.0–34.2)	**0.026**
Platelets (x10^3/μl)	176.0 (70.0–444.0)	175.5 (70.0–444.0)	176.0 (77.0–294.0)	0.85	176.0 (70.0–444.0)	175.5 (77.0–294.0)	0.72

ALP, alkaline phosphatase; ALT, alanine amino transferase; AST, aspartate amino transferase; CCC, cholangiocellular carcinoma; CTx, chemo therapy; GGT, gamma glutamyl transferase; HCC, hepatocellular carcinoma; LD, liver dysfunction; mCRC, metastatic colorectal cancer; PDR, plasma diffusion rate; POD, postoperative day; PT, prothrombin time; R15, retention rate at 15 minutes; SB, serum bilirubin

^a^ PT is expressed in relation to the coagulation time of a healthy person (i.e. Quick). Accordingly, it is illustrated in percentage.

Furthermore, this study involves the analysis of ICG-clearance testing as well as patient data comparison and was approved by the Institutional Ethics Committee of the Medical University of Vienna, in accordance with the Declaration of Helsinki (59th WMA General Assembly, Seoul, Republic of Korea, October 2008). All patients gave written informed consent. Furthermore, the trial was registered at a clinical trials registry (ClinicalTrials.gov Identifier: NCT01700231; NCT02113059).

### ICG—Clearance Test

Perioperative liver function was evaluated by the ICG-clearance testing as previously described [17]. Briefly, pulse spectrometry was used to quantify the patient 's blood ICG concentration. Particularly, a dose of 25mg dye was dissolved in 20ml of distilled water, immediately before injection. The injected amount was based on the body weight ratio of the patient (0.25mg/kg). The two parameters, PDR and R15, were recorded with a Limon—Technology device (Pulsion Medical System SE, Germany) and automatically calculated in accordance with the time course of blood ICG concentration.

### Definition and Classification of Postoperative LD, Morbidity and Length of Stay

The criteria issued by the international study group of liver surgery (ISGLS) were applied to evaluate postoperative LD [18]. Accordingly, LD was defined by an abnormal serum bilirubin level and prothrombin time on or after POD5. At our institution these threshold values are 1.2 mg/dl for serum bilirubin and 75% for prothrombin time (illustrated as Quick). Accordingly, patients exceeding these cut offs (>1.2 mg/dl serum bilirubin and <75% prothrombin time (Quick) were classified as postoperative LD. However, as suggested by the ISGLS criteria, if a patient was suffering from an abnormal serum bilirubin or prothrombin time already prior to surgery, postoperative LD was classified if serum bilirubin or prothrombin time worsened after POD5. Of note, for patients who reached normal serum bilirubin or prothrombin time values prior to POD 5 and were discharged early due to good clinical performance, no further blood collection could be performed on or after POD 5; these patients were considered as “no LD”.

Postoperatively, patients were followed for 90 days and postoperative morbidity was prospectively evaluated. The classification scheme by Dindo et al. [19] was applied and the severity of postoperative complications was classified in grade I to V. In case of multiple complications per patient, the most serious one was graded. According to this classification scheme, all patients requiring surgical, endoscopic or radiological intervention or suffering from life-threatening complications or dying due to postoperative complications, were further defined as “severe morbidity” (grade III-V). Additionally, postoperative length of stay was recorded.

### Statistical Analyses

Statistical analyses were performed using SPSS 20 software (SPSS, Inc., Chicago, IL, USA) and were mainly based on non-parametric tests (Mann-Whitney U test, Wilcoxon test, chi-squared test). To validate the ability of ICG-clearance testing to detect poor postoperative outcome a “receiver operating characteristic” (ROC) curve analysis was performed. In addition to this, this statistical approach was used to identify the optimal cut-off level to distinguish between high and low risk patients. Boxplot illustrations are given without outliers and extreme values to improve the resolution of interquartile ranges. Values were defined as outliers if they exceeded 1.5 till 3 times of the inter quartile range, while they were classified as extreme values if they exceeded more than 3 times of the inter quartile range. P values <0.05 were considered statistically significant.

## Results

### ICG-Clearance Deteriorates After Liver Resection and is Affected by the Extent of Liver Resection

**Fig 1:**
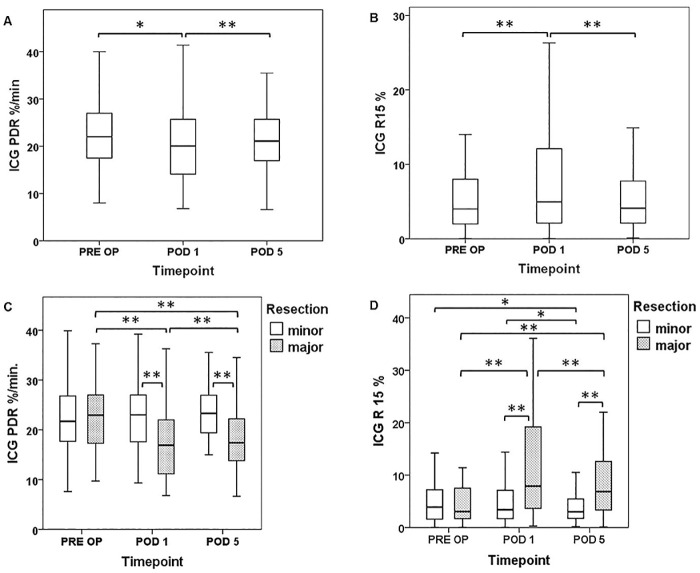
Perioperative ICG-Clearance Time Course. ICG-clearance was measured 1 day prior to surgery (Pre OP), on the first postoperative day (POD 1) and five days (POD 5) after liver resection. The perioperative time course of ICG plasma diffusion rate (PDR [A]) and retention rate after 15 minutes (R15 [B]) is illustrated and shown separately according to the extent of resection (PDR = C, R15 = D). Boxplot illustrations are given without outliers and extreme values to improve the resolution of interquartile ranges. * P <0.05, ** P <0.005.

Since the ICG-clearance test quantitatively reflects the parenchymal function as well as flow conditions of the liver [12, 20], the perioperative time course of ICG-clearance was initially characterized in patients undergoing liver resection (Fig 1A/1B).

Accordingly, perioperative ICG-values revealed a distinct and significant decrease in PDR and an increase in R15 on POD 1 (median PDR: pre OP = 22.1%/min vs. POD 1 = 20.1%/min, P = 0.007; median R15: pre OP = 3.6% vs. POD1 = 5.0%, P = 0.001). PDR and R15 recovered significantly, until POD 5, and almost reached preoperative values (median PDR: POD 1 = 20.1%/min vs. POD 5 = 21.10%/min, P = 0.002; median R15: POD 1: 5.0% vs. POD 5 = 4.10%, P = 0.001).

To evaluate if the extent of liver resection would affect ICG-clearance, PDR and R15 were comparatively analyzed in patients undergoing minor or major resections (Fig 1C/1D). In patients undergoing major resections ICG-clearance was found to be significantly worse than in patients with minor resections on POD 1 (median PDR: major resection = 16.9%/min vs. minor resection = 23.0%/min, P = 0.001; median R15: major resection = 7.9% vs. minor resection = 3.4%, P = 0.001). Although PDR and R15 tended to recover in both groups, this difference persisted till POD 5 (median PDR: major resection = 17.4%/min vs. minor resection = 23.3%/min, P = 0.001; median R15: major resection = 6.9% vs. minor resection = 3.0%, P = 0.001, Fig 1C/1D).

### Preoperative ICG—Clearance is Associated with Poor Clinical Outcome

**Fig 2:**
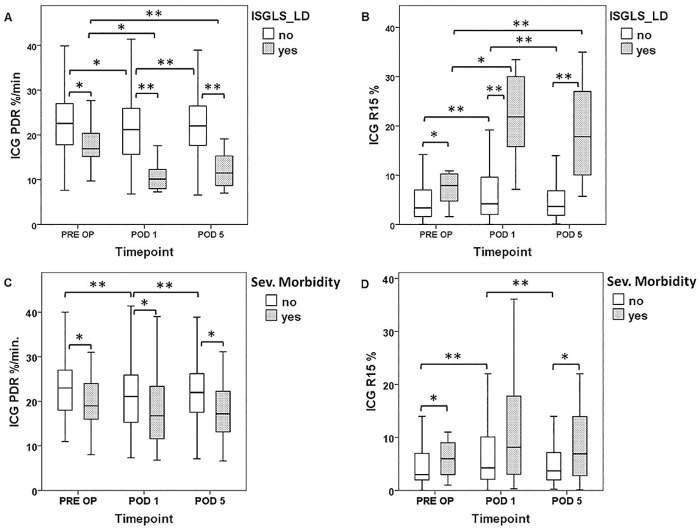
Perioperative ICG-Clearance According to Postoperative LD and Morbidity. ICG (Indocyanine Green)-clearance was measured 1 day prior to surgery (pre OP), on the first postoperative day (POD 1) and on the fifth postoperative day (POD 5). Patients were divided in groups with or without postoperative liver dysfunction (plasma diffusion rate [PDR = A], retention rate at 15 minutes [R15 = B]) and severe morbidity (PDR = C, R15 = D). Boxplot illustrations are given without outliers and extreme values to improve the resolution of interquartile ranges. * P <0.05, ** P <0.005.

**Fig 3:**
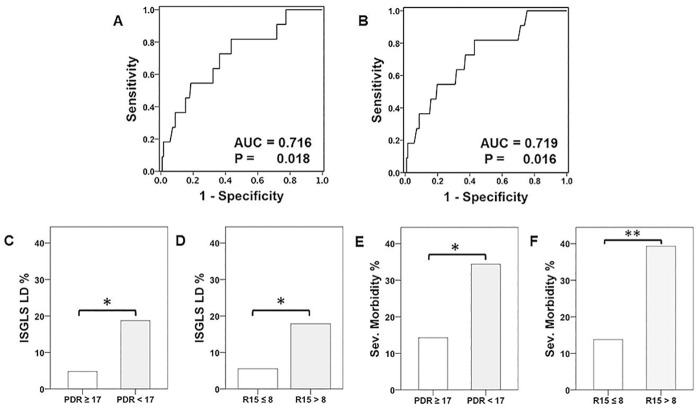
Perioperative ICG—Clearance According to Severity of Postoperative LD. Indocyanine Green (ICG)-clearance was measured 1 day prior to surgery (pre OP), on the first postoperative day (POD 1) and five days after liver resection (POD 5). ICG plasma diffusion rate (PDR [A]) and ICG retention rate at 15 minutes (R15 [B]) are illustrated according to severity of postoperative liver dysfunction (LD) according to International Study Group of Liver Surgery (ISGSL) criteria. * P <0.05, ** P <0.005.

When patients with and or without postoperative LD were further compared, individuals suffering from postoperative LD (11 out of 137) were found to exhibit significantly worse preoperative PDR and R15 values (median PDR: no LD = 22.6%/min vs. LD = 16.9%/min, P = 0.018; median R15: no LD = 3.4% vs. LD = 7.9%, P = 0.016). The perioperative time course of ICG clearance according to postoperative LD is illustrated in Fig 2(A)/2(B) and graded according to severity of LD in Fig 3. Furthermore, specific preoperative characteristics of patients were prospectively recorded and are compared according to the occurrence of postoperative LD as well as with respect to the extent of liver resection (Table 3)

**Table 3:** Pre-OP Levels (LD & Hepatic Resection), Patient demographics, outcome- and laboratory parameters.

Parameters	Collective (N = 137)						
	Pre OP	No LD	LD	P—value	Minor Resection	Major Resection	P—value
	N = / Median (Range/ %)	N = / Median (Range/ %)			N = / Median (Range/ %)		
**Age**	63 (22–89)	63 (22–89)	64 (38–81)	0.42	62 (22–85)	64 (23–89)	0.65
**Sex**							
Male	88 (64.2%)	79 (62.7%)	9 (81.8%)	0.21	57 (74.0%)	31 (51.7%)	**0.007**
Female	49 (35.8%)	47 (37.3%)	2 (18.2%)		20 (26.0%)	29 (48.3%)	
**Neoplastic entity**							
mCRC	59 (43.1%)	58 (46.0%)	1 (9.1%)	0.13	37 (48.1%)	22 (36.7%)	0.06
HCC	30 (21.9%)	26 (20.6%)	4 (36.4%)		14 (18.2%)	16 (36.7%)	
CCC	23 (16.8%)	19 (15.1%)	4 (36.4%)		8 (10.4%)	15 (35.0%)	
Non neoplastic Adenomas or Haemangiomas	13 (9.5%)	12 (9.5%)	1 (9.1%)		9 (11.7%)	4 (6.7%)	
Others	12 (8.8%)	11 (8.7%)	1 (9.1%)		9 (11.7%)	3 (5.0%)	
**Preoperative CTx**							
Yes	51 (37.2%)	50 (40.0%)	1 (9.1%)	**0.042**	27 (35.5%)	24 (40.0%)	0.59
**Hepatic Resection**							
Major	60 (43.8%)	50 (39.7%)	10 (90.9%)	**0.001**			
Minor	77 (56.2%)	76 (60.3%)	1 (9.1%)				
**Child Pugh Score/ Missing values (N = 19)**							
A	109 (92.4%)	102 (93.6%)	7 (77.8%)	0.09	63 (96.9%)	46 (86.8%)	**0.039**
B	9 (7.6%)	7 (6.4%)	2 (22.2%)		2 (3.1%)	7 (13.2%)	
MELD Score	6 (6–16)	6 (6–16)	7 (6–12)	0.17	6 (6–12)	6 (6–16)	0.86
**Preoperative Parameters**							
PDR %/min.	22.1 (7.6–42.8)	22.6 (7.6–42.8)	16.9 (9.7–27.7)	**0.018**	21.7 (7.6–42.8)	23.0 (9.7–37.3)	0.97
R15%	3.6 (0.0–32.0)	3.4 (0.0–32.0)	7.9 (1.6–23.3)	**0.016**	4.2 (0.0–32.0)	3.1 (0.0–23.3)	0.59
SB mg/dl	0.6 (0.2–6.6)	0.6 (0.2–3.2)	0.9 (0.5–6.6)	0.13	0.6 (0.2–2.9)	0.6 (2.7 (6.6)	0.88
PT % ^a^	102.5 (40.0–150.0)	103.0 (40.0–150.0)	100.0 (78.0–150.0)	0.64	102.5 (61.0–150.0)	103.5 (40.0–150.0)	0.84
ALP U/l	86.0 (14.0–946.0)	86.0 (14.0–946.0)	96.0 (64.0–396.0)	0.47	84.0 (14.0–165.0)	98.5 (51.0–946.0)	**0.002**
GGT U/l	58 (11–699)	54 (11–699)	92 (33–386)	**0.047**	47 (12–278)	69 (11–699)	**0.021**
AST U/l	29.0 (14.0–208.0)	29.0 (14.0–175.0)	41.0 (21.0–208.0)	**0.040**	27.0 (14.0–175.0)	32.0 (16.0–208.0)	**0.005**
ALT U/l	26.0 (6.0–196.0)	25.0 (6.0–196.0)	41.5 (21.0–120.0)	**0.020**	24.5 (10.0–196.0)	32.0 (6.0 167.0)	0.22
Albumin g/l	42.2 (32.5–52.4)	42.3 (3.1–52.4)	41.1 (32.5–47.2)	0.33	42.2 (32.5–52.4)	42.1 (33.0–47.3)	0.14
Platelets (x10^3/μl)	226.5 (92.0–492.0)	227.5 (92.0–492.0)	217.0 (152.0–303.0)	0.72	220.0 (103.0–470.0)	235.0 (92.0–492.0)	0.07
Creatinine mg/dl	0.9 (0.5–1.9)	0.8 (0.5–1.9)	0.9 (0.7–1.4)	0.39	0.9 (0.5–1.8)	0.8 (0.5–1.9)	**0.019**
INR	0.9 (0.7–1.8)	0.9 (0.7–1.8)	1.0 (0.7–1.1)	0.30	0.9 (0.7–1.3)	0.9 (0.7–1.8)	0.57

ALP, alkaline phosphatase; ALT, alanine amino transferase; AST, aspartate amino transferase; CTx, chemo therapy; GGT, gamma glutamyl transferase; INR, international normalized ratio; LD, liver dysfunction; MELD, model of end-stage liver diseases, PDR, plasma diffusion rate; preOP, preoperative; PT, prothrombin time; R 15, retention rate at 15 minutes; SB, serum bilirubin

^a^ PT is expressed in relation to the coagulation time of a healthy person (i.e. Quick). Accordingly, it is illustrated as the percentage of the normal value of the healthy population.

Similarly, preoperative ICG-clearance was significantly worse in patients suffering from severe postoperative morbidity (26 out of 137; median PDR: no severe morbidity = 22.6%/min vs. severe morbidity = 19.3%/min, P = 0.013; median R15: no severe morbidity = 3.4% vs. severe morbidity = 5.5%, P = 0.035). The perioperative time course of ICG clearance parameters according to severe postoperative morbidity is illustrated in Fig 2C/2D.

### Postoperative ICG-Clearance allows Early Postoperative Prediction of LD and Clinical Outcome

Since there is no uniform consensus on the predictive potential of postoperative ICG—clearance [7, 10, 11, 21–23] postoperative PDR and R15 levels were further evaluated for their association with clinical outcome. Indeed, patients suffering from postoperative LD were found to have significantly worse PDR and R15 levels in the early postoperative period (POD 1) as well as persistently inferior values until POD 5 after LR (POD 1: no LD median PDR = 21.2%/min vs. LD median PDR = 10.2%/min, P = 0.001; no LD median R15 = 4.2% vs. LD median R15 = 21.9%, P = 0.001; POD 5: no LD median PDR = 22.0%/min vs. LD median PDR = 11.5%/min, P = 0.001, no LD median R15 = 3.7% vs. LD median R15 = 17.8% P = 0.001). The perioperative time course in relation to postoperative LD is illustrated in Fig 2(A)/2(B) and graded according to severity in Fig 3.

Similarly, patients suffering from severe postoperative morbidity were found to have inferior PDR and R15 values on POD 1, which also failed to recover, as reflected by a lasting alteration of these parameters until POD 5 (POD 1: no severe morbidity median PDR = 21.1%/min vs. severe morbidity median PDR = 16.8%/min, P = 0.047, no severe morbidity median R15 = 4.3% vs. severe morbidity median R15 = 8.2%, P = 0.051; POD 5: no severe morbidity median PDR = 22.0%/min vs. severe morbidity median PDR = 17.2%/min, P = 0.008, no severe morbidity median R15 = 3.7% vs. severe morbidity median R15 = 6.9%, P = 0.027). The postoperative time course according to severe morbidity is illustrated in Fig 2(C)/2(D).

### Preoperative ICG-Clearance is a Significant Predictor of Postoperative LD and Severe Postoperative Morbidity

**Fig 4:**
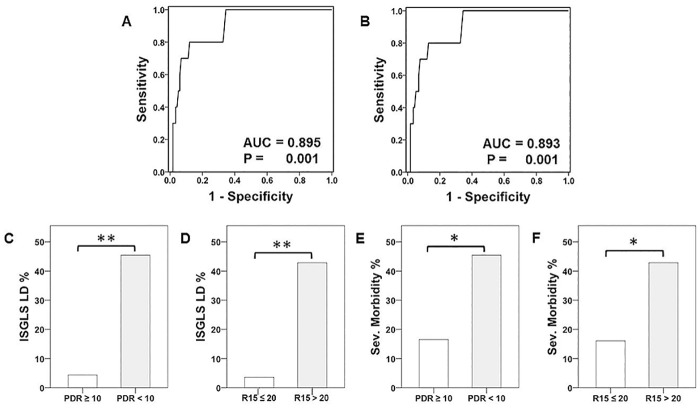
Preoperative ICG-Clearance and Prediction of Postoperative Outcome. Receiver operating characteristic (ROC)- curve analysis for preoperative ICG (Indocyanine Green)–clearance to predict postoperative liver dysfunction (LD) is illustrated (plasma diffusion rate [PDR = A], retention rate at 15 minutes [R15 = B]). The incidence of postoperative LD (PDR = C, R15 = D) as well as of severe postoperative morbidity (PDR = E, R15 = F) is shown according to the defined cut-off values for preoperative ICG-Clearance (PDR <17, R15 >8). * P <0.05, ** P <0.005.

The potential of preoperative ICG-clearance to predict postoperative LD was further evaluated. Therefore, a ROC curve analysis was performed, revealing a significant predictive value of PDR (area under the curve (AUC): pre OP: 0.716, P = 0.018, Fig 4A) and R15 (AUC: pre OP: 0.719, P = 0.016; Table 4, Fig 4B). Using ROC analysis, a preoperative cut-off level of PDR <17%/min and R15 >8% was chosen to specifically identify patients with postoperative LD and severe morbidity. To evaluate whether unfavorable preoperative PDR or R15 values would further translate into poor clinical performance, the incidence of postoperative LD and severe morbidity was compared. Accordingly, patients fulfilling the newly defined cut-offs were found to suffer from an increased incidence of postoperative LD and severe morbidity. Additionally, postoperative hospitalization was prolonged in patients with deteriorated ICG—values (Table 5, Fig 4C–4F).

**Table 4:** Perioperative ICG-Clearance.

	**Preoperative**		**POD 1**	
**LD**	**PDR ≥/< 17%/min.**	**R15 >/≤ 8%**	**PDR≥/< 10%/min.**	**R15 >/≤ 20%**
Specificity (%)	79.4	81.8	94.8	93.0
Sensitivity (%)	54.6	45.5	50.0	60.0
NPV	95.2	94.5	95.7	96.4
PPV	18.8	17.9	45.5	42.9
	**Preoperative**		**POD 1**	
**Severe Morbidity**	**PDR ≥/< 17%/min.**	**R15 >/≤ 8%**	**PDR ≥/ < 10%/min.**	**R15 >/≤ 20%**
Specificity (%)	80.9	84.6	94.1	92.2
Sensitivity (%)	42.3	42.3	20.8	25.0
NPV	85.6	86.1	83.5	83.9
PPV	34.4	39.3	45.5	42.9

LD, liver dysfunction; NPV, negative predictive value; PDR, plasma diffusion rate; POD, postoperative day; PPV, positive predictive value; R 15, retention rate at 15 minutes

**Table 5:** Clinical Outcome Parameters.

	LD (%)	P-value	Severe morbidity (%)	P-value	Hospitalisation (days)	P-value
**PRE OP**						
**PDR > 17%/min.**	4.8	**0.011**	14.4	**0.012**	8	**0.028**
**PDR < 17%/min.**	18.8		34.4		10	
**R15 <8%**	5.5	**0.032**	13.9	**0.002**	8	**0.041**
**R15 > 8%**	17.9		39.3		10	
**POD 1**						
**PDR > 10%/min.**	4.3	**0.001**	16.5	**0.020**	8	**0.022**
**PDR < 10%/min.**	45.5		45.5		12	
**R15 < 20%**	3.6	**0.001**	16.1	**0.016**	7	**0.019**
**R15 >20%**	42.9		42.9		11	

LD, liver dysfunction; PDR, plasma diffusion rate; POD, postoperative days; Pre OP, preoperative; R15, retention rate at 15 minutes

Furthermore, the potential of commonly used scores of liver function to predict postoperative clinical outcome was evaluated. Accordingly, no significant associations between preoperative MELD- or Child Pugh Score levels and severe postoperative complications (i.e. LD) could be observed (LD: MELD, P = 0.17; Child Pugh, P = 0.09; Table 3). Moreover, patients receiving major resections had a higher prevalence of Child Pugh B (Child B: minor = 3.1%, major = 13.2%; P = 0.039; Table 3). In addition, specific characteristics of patient´s MELD- and Child Pugh Score values were compared according to ICG-clearance cut-off levels and are listed in Tables 1 and 2, revealing that patients who fulfilled our preoperative cut-off values suffered from significantly higher MELD score levels (PDR: median ≥17 = 6 vs. <17 = 7, P = 0.014; R15: median ≤8 = 6, >8 = 7, P = 0.047). No significant differences between postoperative ICG cut off- and MELD score- levels could be observed. Furthermore, when looking at possible correlations between Child Pugh score and ICG cut-off values, only postoperative, not preoperative deteriorated ICG values where associated with Child B (Child B: PDR: ≥10 = 6.2% vs. <10 = 27.3%, P = 0.016; R15: ≤20 = 6.3%, >20 = 23.1%), P = 0.040).

### Postoperative ICG-Clearance is a Significant Predictor of Postoperative LD and Severe Postoperative Morbidity

**Fig 5:**
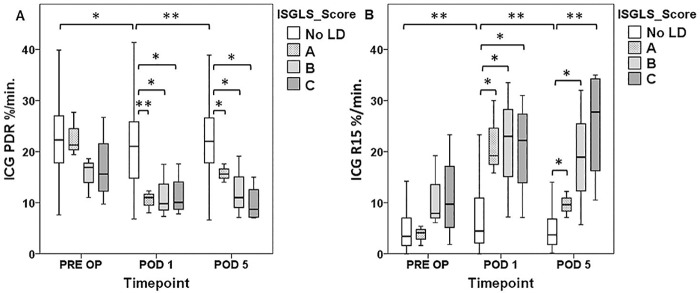
Postoperative ICG—Clearance and Prediction of Postoperative Outcome. Receiver operating characteristic (ROC)- curve analysis for ICG (Indocyanine Green)–clearance on postoperative day one (POD 1) to predict postoperative liver dysfunction (LD) is illustrated (plasma diffusion rate [PDR] = A, retention rate at 15 minutes [R15] = B). The incidence of postoperative LD (PDR = C, R15 = D,) as well as of severe postoperative morbidity (PDR = E, R15 = F) is shown according to the defined cut-off values for ICG-clearance on POD 1 (PDR <10, R15 >20). * P <0.05, ** P <0.005.

To further evaluate the predictive potential of postoperative assessed ICG—clearance, a ROC curve analysis was performed. Accordingly, postoperative PDR and R15 were found to be significantly associated with postoperative LD (POD 1: PDR-AUC = 0.895, P = 0.001; R15-AUC = 0.893, P = 0.001; Table 4, Fig 5A/5B). As expected, basic characteristics of these groups differed significantly with an increased prevalence of major LR and worse postoperative liver function parameters (Table 2). Again, ROC analysis was used to define suitable cut-off levels: PDR <10%/min., R15 >20%. Incidences of postoperative LD and severe morbidity as well as postoperative hospitalization were increased as illustrated in Table 5 and Fig 5(C)–5(F).

## Discussion

Consecutively including all patients undergoing hepatic resection, this study used a clinically relevant setting to evaluate the potential of perioperative ICG-clearance to predict postoperative LD and morbidity, as defined by highly standardized and well established criteria. Accordingly, the investigators were able to demonstrate that preoperative ICG-clearance testing was significantly associated with postoperative LD and severe morbidity. Furthermore, postoperative ICG-clearance testing was able to detect poor postoperative outcome as early as on the first post-operative day. For both, pre- and post-operative ICG-clearances, cut—off values were defined, suitable for a Western population, to specifically identify low risk patients that are unlikely to develop postoperative LD or complications. In contrast, patients exceeding the newly defined cut-offs should undergo a careful reevaluation of their treatment options.

The definition of postoperative LD has been under debate during the past few years. As varying definitions of post-hepatectomy LD were used, comparability between studies is difficult. The ISGLS has put great effort in defining a clinically relevant score to reflect postoperative LD in patients undergoing liver resection [18]. Accordingly these standardized criteria were used to define postoperative LD within this study. Based on this grading system; a distinct evaluation of the predictive potential of preoperative ICG-clearance for postoperative LD was performed. Furthermore, to achieve highest comparability and standardization, the Dindo-Clavien classification was used to define and specify postoperative morbidity in the investigated patients collective [19]. As for postoperative LD, preoperative ICG-clearance was able to predict postoperative severe morbidity, thereby demonstrating the clinical relevance of preoperative ICG-clearance measurement.

While several previous studies reported comparable results, [13–15, 24, 25] the reported results are in part contradictory to previous reports. Very recently, Wong et al. were unable to document a predictive value of R15 in their high-risk patients, despite using the same methodology for ICG-clearance testing and the same definition of postoperative severe morbidity [6]. The fairly high rate of viral hepatitis and the low rate of alcoholic as well as non-alcoholic fatty liver disease in the collective of Wong et al. compared to the present study population (a Western collective) might account for this difference [6]. Indeed, probably due to these differences preoperative ICG R15 levels were also much higher compared to the baseline R15 values within our Western patient cohort (Wong et al.: median = 17.6% [range = 4.2%–54.9%] vs. 3.6% [range = 0.0–32.0]) [6].

Little is known about the relevance of ICG-clearance testing in a Western setting. In particular, in this study 43.1% of patients suffered from metastatic colorectal cancer and almost 37% of all patients received preoperative chemotherapy, which substantially differs from a classical Eastern collective [8, 13]. Preoperative liver function assessment in these patients is of particular importance, as neoadjuvant chemotherapy is known to induce sinusoidal obstruction syndrome as well as steatohepatitis and does thereby affect ICG-clearance [17, 21]. Furthermore, the incidence of obesity is substantially higher in the Western population, which has been shown to reduce the accuracy of preoperative liver function assessment [26].

Importantly, despite these differences, this study was able to demonstrate that perioperative ICG-clearance is associated with postoperative clinical outcome.

According to the results of this investigation, clinically relevant cut-offs for PDR (preoperative >/< 17; POD 1 >/< 10) as well as R15 (preoperative >/< 8; POD 1 >/< 20) before and after liver resection could be defined. The combination of both parameters did not increase the predictive potential of ICG—clearance (data not shown). Despite the theoretical association that R15 is a direct function of PDR, we observed that R15 and PDR values are different when measured with the Pulsion detector (Limon—Technology device, Pulsion Medical System SE, Germany). However, patients who were suffering from preoperative deteriorated PDR values (<17%/min.) concomitantly showed higher R15 values too (>8%). Similar circumstances were observed when looking at our postoperative PDR and R15 cut-off levels (data not shown). Nevertheless, in a clinical routine setting, both, abnormal PDR or R15 values, should result in a careful reevaluation of the patient`s operability.

In contrast to ICG-clearance testing, no association of conventional liver function assessments, such as MELD- or Child Pugh Score, with postoperative clinical outcome was observed. This might suggest that ICG-clearance is comparably more sensitive than conventional liver functions assessments to predict postoperative clinical outcome. However, also using ICG clearance testing, some patients suffering from postoperative LD and severe morbidity were not identified. In fact, the negative predictive values of PDR and R15 were specifically high (>80%), while the positive predictive values ranged around 30%. Therefore, ICG-clearance seems specifically helpful to identify patients that will not develop postoperative LD or severe morbidity. Accordingly, there is still an urgent need to further improve preoperative liver function assessment in the remaining “high-risk” patients. Additional liver function assessments might further improve the preoperative risk stratification. While dynamic tests have shown very promising results [5–9], also circulating blood parameters, such as intra-platelet serotonin might help to allow for a more detailed preoperative risk stratification in the near future [27]. However, these parameters still need to be validated in large-scale, prospective clinical trials.

Quantifying postoperative liver function still remains a difficult task in liver surgery. While classical liver function parameters might be largely affected by the intra-operative course, postoperative ICG—clearance represents an attractive option to determine postoperative liver function reserve [15]. Despite the fact that ICG-clearance is affected by postoperative hemodynamics, [28] ICG-clearance on POD 1 was found to be strikingly associated with postoperative LD and severe morbidity. Therefore, ICG-clearance on POD 1 seems to represent a useful parameter to identify patients who require close monitoring for potential complications and suitable interventions. In particular, these patients might benefit from additional radiological diagnostic evaluation and from close monitoring of blood parameters to allow early detection of postoperative complications. A postoperative ICG- clearance exceeding the newly defined cut offs might enable an early transfer to the intensive care unit and for immediate antibiotic therapy, if indicated, since patients with LD are known to be more susceptible to infections. Furthermore, the timely initiation of liver support devices might be guided by postoperative ICG-clearance testing.

## Conclusion

Taken together, perioperative ICG-clearance was found to be a valuable predictor of LD, severe morbidity and prolonged hospitalization after liver resection in a Western clinical setting using standardized outcome parameters. ICG-clearance is non-invasive as well as easily assessable and therefore represents a useful clinical parameter to distinguish low- and high-risk patients prior to as well as after surgery who require consideration and close monitoring for potential complications.
